# Therapeutic effect of liposomal prostaglandin E_1_ in acute lower limb ischemia as an adjuvant to hybrid procedures

**DOI:** 10.3892/etm.2013.1061

**Published:** 2013-04-10

**Authors:** JIANLIN LI, BING WANG, YUE WANG, FEI WU, PANFENG LI, YANG LI, LEI ZHAO, WENJUN CUI, YU DING, QIAN AN, JIANGTAO SI

**Affiliations:** Department of Vascular Surgery, The Fifth Affiliated Hospital of Zhengzhou University, Zhengzhou, Henan 450052, P.R. China

**Keywords:** liposomal prostaglandin E1, acute lower limb ischemia, hybrid procedures

## Abstract

Prostaglandin E_1_ (PGE_1_) is widely used in the treatment of limb ischemia for its potent vasodilatory and antiplatelet effects. In order to assess the curative effect of liposomal PGE_1_ (lipo-PGE_1_) as an adjuvant to surgery in patients with acute lower limb ischemia (ALLI), 204 patients who underwent hybrid procedures (operative thromboembolectomy or bypass and necessary endovascular interventions) for ALLI were randomly divided into a blank control group and a lipo-PGE_1_ group (intravenous infusion of 20 *μ*g/day for 12–14 consecutive days following surgery). Patients were followed-up for 6 months after surgical revascularization for clinical events. The primary study endpoint, which was the combined incidence of perioperative (30 days) mortality (POM) and major adverse limb events (MALE; amputation or major intervention), was significantly reduced in patients treated with lipo-PGE_1_ (5.1% compared with 13.2% in the control group). The overall incidence of clinical events, including POM, MALE and major adverse cardiovascular events, was significantly reduced in patients receiving lipo-PGE_1_ (8.2%) compared with the controls (20.8%). Hybrid procedures are an improved method for treating ALLI and may remedy underlying lesions of vessels following thromboembolectomy.

## Introduction

Over the past decade, the treatment of acute lower limb ischemia (ALLI) has become more complicated, largely due to the decline in the number of patients presenting with embolism owing to rheumatic vascular disease and atrial fibrillation and, conversely, a sharp increase in elderly people with advanced atherosclerosis presenting with thrombosis and a far more complex disease pattern. Patients presenting with ALLI require persistently high levels of medical care, yet show poor clinical outcomes with high amputation and mortality rates, despite improvements having been made in surgical techniques and perioperative patient care (1). Prostaglandin E_1_ (PGE_1_) has been widely used in the treatment of peripheral vascular disease (2) due to its various pharmacological activities, including potent vasodilation, inhibition of leukocyte adhesion and platelet aggregation, and anti-inflammatory activity (3). However, Brass *et al* identified that PGE_1_ failed to improve the outcome of lower limb ischemia (4). Gabriel *et al* reported that PGE_1_ worsens already impaired cellular oxygen supply (5). Incorporating PGE_1_ into lipid microspheres, as a lipid emulsion of PGE_1_ (lipo-PGE_1_), provides a long duration of action, few side-effects (2) and greater variety of pharmacological effects compared with free PGE_1_(6). Surgical methods of treatment are considered to play a critical role in improving the clinical outcomes of patients with ALLI (7). Hybrid procedures have been demonstrated to provide an improved solution for the treatment of severe peripheral vascular disease (8,9). The present study was designed to assess the therapeutic effects of lipo-PGE_1_ used in patients with ALLI as an adjuvant to hybrid procedures.

## Patients and methods

### Patients

**Figure 1:**
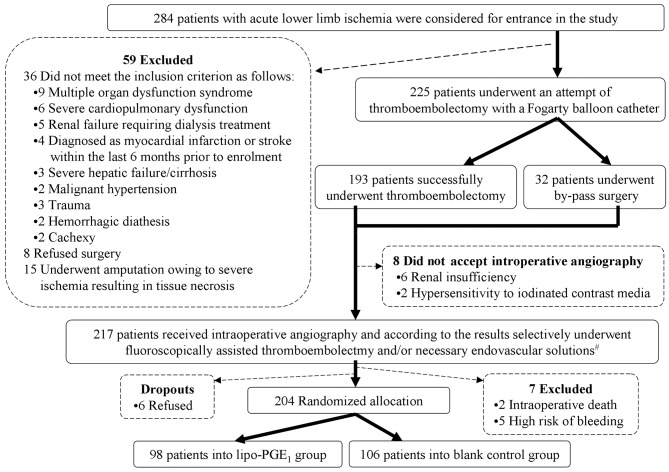
Hybrid procedures used for treating acute lower limb ischemia (ALLI). ^#^Endovascular solutions consisted of balloon angioplasty or/and stenting.

This study was performed according to the Declaration of Helsinki and the protocol was approved by the ethics committee of Zhengzhou University (Zhengzhou, China). The patients were considered for enrolment in the study if they presented with acute onset (<14 days) of ischemic symptoms of the legs and were to be treated with hybrid procedures. There were 204 patients (males, 115; females, 89) who met the criteria (Fig. 1).

All patients received a hypodermic injection of 2,500 units low-molecular-weight heparin calcium twice a day for one week after surgery and also received relevant treatment for concomitant diseases, including hypotensor drugs, hypoglycemic agents and lipid-lowering medicines. Commonly used drugs for concomitant clinical conditions were as follows: i) cardiovascular drugs (benazepril, perindopril, valsartan, telmisartan, losartan, extended-release nifedipine, amlodipine besylate, levamlodipine besylate, extended-release felodipine, isosorbide mononitrate, nitroglycerin, indapamide, chlorthalidone and metoprolol tartrate); ii) hypoglycemic drugs (insulin, glimepiride, gliclazide, sitagliptin, metformin and acarbose); iii) statins (atorvastatin, simvastatin and rosuvastatin); and iv) antineoplastic drugs (carboplatin, rituximab, vincristine, floxuridine and etoposide). The dosage and category of individual drugs was adjusted according to the suggestions of the consultants. Patients were instructed to take 2.5 mg/day warfarin from the third day after surgery; thus the overlapping time between low-molecular-weight heparin calcium administration and warfarin administration was 3–4 days. The blood coagulation function was detected every three days after having taken warfarin for three days, and the dosage of warfarin was adjusted according to the international normalized ratio (INR)results, until the INR indicated a therapeutic effect (2–3). When patients took warfarin, aspirin and thienopyridines were not administered.

### Interventions

The 204 patients were randomly divided into a lipo-PGE_1_ group and blank control group (Table I). Following surgery, the lipo-PGE_1_ group received a daily 6 h intravenous infusion of lipo-PGE_1_ (Kaishi, Beijing Tide Pharmaceutical Co., Ltd., Beijing, China) at a dose of 20 *μ*g/day for 12–14 consecutive days. Throughout the study, treatment with other vasoactive drugs, including buflomedil, fasudil and iloprost were not permitted. All patients were followed up for 6 months for occurrence of major clinical events. During the study period, the combined incidence of perioperative (30 days) mortality (POM) and major adverse limb events (MALE) was defined as the primary endpoint. The secondary efficacy endpoint was defined as the occurrence of a major adverse cardiovascular event (MACE), which consisted of myocardial infarction, stroke or mortality from any cause (10). During the follow-up period, the concentration of hemoglobin A1C of diabetic patients was tested every three months if the patient had stable glycemic control; otherwise it was tested once a month. The target level for low-density lipoprotein (LDL)-cholesterol was <100 mg/dl for the majority of patients and was expected to be <70 mg/dl or a reduction of 30–40% from the baseline for patients having diabetes and/or cardiovascular disease. The level of LDL-cholesterol of patients taking statins was measured once a month.

### Statistical analysis

The unpaired t-test and Chi-square test or Fisher’s exact test were employed for comparison of the baseline results of the two groups, as appropriate. The clinical outcomes (POM, combined POM and MALE and all clinical events) of the two groups were evaluated with the log-rank test. Multivariable analyses were performed with a Cox proportional hazard regression model to compare the outcomes for experimental treatment, class of ischemia according to SVS-ISCVS-TASC criteria (1,11) (class I-IIa vs. IIb-III) and surgical method (thromboembolectomy vs. bypass). Proportional hazard assumptions were tested for all the covariates. All statistical tests were two-sided and a value of P<0.05 was considered to indicate a statistically significant difference. Statistical analyses were performed using SPSS software (version 12.0, SPSS, Inc., Chicago, IL, USA) on the basis of the intention-to-treat analysis.

## Results

**Figure 2:**
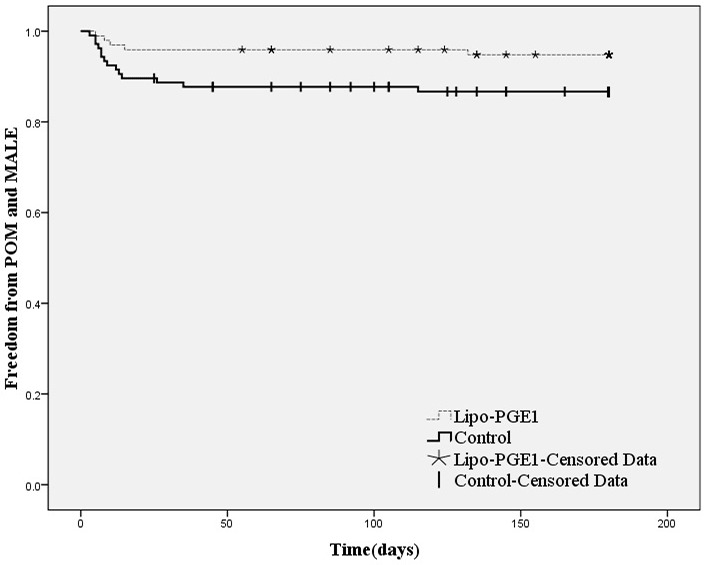
Kaplan-Meier estimates of freedom from perioperative mortality (POM) and major adverse limb events (MALE) in the two study groups. Lipo-PGE_1_, liposomal prostaglandin E_1_.

In the control and lipo-PGE_1_ group, 8.5 and 6.1% of patients were lost to follow-up, respectively. Due to the inconvenience of measuring blood coagulation or poor compliance, 42.6% patients received aspirin and clopidogrel (antiplatelet therapy) instead of warfarin during the follow-up period, and only 8 patients (5 patients in the control group and 3 patients in the lipo-PGE_1_ group) stopped taking oral anticoagulants and anti-platelet drugs 1–2 months after being discharged from hospital. The treatment for concomitant diseases were similar for the two groups (statins, 71.7 and 74.5%; anti-hypertensive drugs, 56.6 and 58.2%; warfarin, 45.3 and 46.9%; and antiplatelet therapies, 41.5 and 43.9% in the control and lipo-PGE_1_ groups, respectively). The lipo-PGE_1_-treated and control groups did not differ with respect to baseline characteristics (Table I). The comparative clinical outcomes of the two groups are shown in Table II. The combined incidence of POM and MALE (primary end-point) was significantly lower in the patients receiving lipo-PGE_1_ than in the controls. The Kaplan-Meier curves for freedom from POM and any MALE are shown in Fig. 2. The overall incidence of all clinical events (POM+MALE+MACE) was significantly reduced in the lipo-PGE_1_ patients compared with the control group. Multivariable analysis revealed that the occurrence of POM or MALE was less frequent for patients with ischemia of class I-IIa (compared with IIb-III) and this was more related to bypass procedures (compared with thromboembolectomy procedures; Table III). No serious adverse reactions occurred following lipo-PGE_1_ administration.

## Discussion

Previous studies have reported a 30-day amputation rate of 5–12% and mortality risk of 9.9–42% for patients with ALLI (12–15). The risk factors that may lead to poor prognosis include the absorption of metabolic toxins associated with acute ischemia, ischemia-reperfusion injury and the incomplete restoration of perfusion (residual thrombi in distal vessels not reached by the Fogarty catheter thromboembolectomy, propagation of residual thrombus or presence of underlying steno-occlusive lesions). Pemberton *et al* demonstrated that, following thromboembolectomy, 24% patients required an immediate further procedure and 15% patients underwent further limb salvage surgery within 30 days (15). In the present study, the combined incidence of POM and MALE in the two groups (13.2 and 5.1% in the lipo-PGE_1_ and control groups, respectively) was far lower than previous reports, which may demonstrate the benefit of hybrid procedures. Intraoperative completion angiography allowed the surgeons to discern the defects of vessels following surgical revascularization and guided them to repair lesions by means of endovascular techniques, which may improve the primary patency (16). Bosma and Jörning identified using angiography that following thromboembolectomy, 30% of patients presented incomplete clearance of the arterial tree, resulting in a requirement for further embolectomy, as well as a reduced rate of amputation (17). In another study, the adoption of routine intraoperative angiography following thromboembolectomy confirmed that 53.4% patients required intraoperative re-interventions for residual lesions with the result that 2-year primary patency rates were improved (18). Through intraoperative angiography, we identified a residual thrombus in 30.4% of patients, and 35.3% patients required additional solutions (repeated thromboembolectomy and/or endovascular interventions). These therapeutic strategies are reliable methods for ensuring the patency of the whole arterial tree.

In the present study, lipo-PGE_1_ was used in emergency conditions and not in patients with chronic diseases or as an adjuvant therapy to organ transplantation or elective surgery, as in previously reported studies (19–21). The patients who received intravenous lipo-PGE_1_ presented higher MALE-free and survival rates than the controls. This may be due to the various pharmacological effects of lipo-PGE_1_, which is known to interfere with inflammatory responses and reduce systemic damage following ischemia and reperfusion. The actions of lipo-PGE_1_ include anti-inflammatory activity, reduction of free radicals and cytokine production, protection against ischemia reperfusion injury, improvement of endothelial function, reduced expression of intercellular adhesion molecules, vasodilation, antiplatelet activity, antithrombotic activity and thrombolytic activation, improvement of the microcirculation and amelioration of the rheological property of the blood (19–24). The pharmacological effects may be conducive to dissolving residual thrombi and thrombi in runoff and branch vessels inaccessible to the balloon catheter, as well as increasing blood flow and reducing the incidence of POM and MALE. Lipo-PGE_1_ also has certain visceral protective effects, including reduction of hepatic injury and myocardial infarction size and improvement of lung function (19,24,25), which may help to reduce the occurrence of MACE. Lipo-PGE_1_ as an adjuvant to surgery greatly reduces the incidence of adverse clinical events.

Thromboembolectomy procedures result in a better prognosis than bypass procedures; however, the detailed mechanism is not yet known. A possible explanation may be that bypass procedures as a secondary choice are adopted when the Fogarty catheter is not able to traverse the occluded segment, which may indicate serious local vessel lesions or that a longer vessel is involved. The other possible reasons include anastomotic stricture, changes of blood flow direction resulting in shear stress and intimal hyperplasia. Although the majority of patients (>60% patients in the two groups) presented acute ischemia classified as grade IIb-III or even irreversible (54.9%), the high survival and MALE-free rates support a more aggressive treatment strategy in patients with severe peripheral ischemia; a similar conclusion was made by Mohler *et al*(26).

The current study also presents several limitations, including the necessity of a longer-term follow-up survey and the absence of a placebo group due to the clinical gravity of the patients. Further studies are required to confirm our data and reveal the mechanism by which lipo-PGE_1_ reduces the incidence of adverse clinical events.

Hybrid procedures may improve the clinical outcomes of patients with ALLI by permitting the identification of inflow, outflow, conduit and anastomotic defects intraoperatively and remedying the underlying lesions of vessels. Lipo-PGE_1_ as an adjuvant to surgical revascularization in ALLI significantly lowered the combined incidence of POM and MALE. Further data and studies are required to support the results.

## Figures and Tables

**Table I:** Clinical baseline characteristics of the two groups.

Parameter	Control (n=106)	Lipo-PGE_1_ (n=98)
Age (mean ± SD, years)	64.6±9.4	66.1±8.9
Gender (male/female)	60/46	55/43
Risk factors		
Hypertension	69.8	71.4
Coronary artery disease	26.4	24.5
Diabetes mellitus 2	35.0	37.8
Hemoglobin A1C (mean ± SD)	7.7±1.0	7.9±1.1
Re-evaluation (mean ± SD)	6.7±0.5	6.6±0.6
Hyperlipidemia	58.5	63.3
LDL-cholesterol (mean ± SD, mg/dl)	126±20	123±24
Re-evaluation (mean ± SD, mg/dl)	85±18	89±23
Cerebrovascular disease	25.5	23.5
Atrial fibrillation/arrhythmias	51.9	53.1
Renal disease	14.2	16.3
Chronic peripheral arterial disease	21.7	18.4
Previous revascularization lower limb	17.9	15.3
Carcinoma	16.0	14.3
Smoking (>15 cigarettes/day for >15 years)	39.6	41.8
Still smoking	32.1	31.6
Vessels involved		
Common/external iliac artery	20.8	21.4
Common/superficial femoral artery	71.7	74.5
Popliteal/infrapopliteal artery	7.5	4.1
Clinical category of acute ischemia		
Class I-IIa	33.0	35.7
Class IIb-III	67.0	64.3
Thromboembolectomy (success cases/n)	84.9	86.7
Bypass	15.1	13.3
Adjunctive intervention (%)		
Fluoroscopically assisted thromboembolectomy	20.8	21.4
Balloon angioplasty	13.2	11.2
Stenting	9.4	8.2
Endarterectomy	11.3	10.2
Hospital stay (mean ± SD, days)	15.2±3.4	14.8±3.1

Data are presented as % unless otherwise stated. Lipo-PGE_1_, liposomal prostaglandin E_1_; SD, standard deviation; LDL, low-density lipoprotein.

**Table II:** Outcomes at the 6-month follow-up.

Event	Control	Lipo-PGE_1_
POM	8 (7.5)	3 (3.1)
Major endpoints (POM+MALE)	14 (13.2)	5 (5.1)^a^
MALE	6 (5.7)	2 (2.0)
POM+MALE+MACE	22 (20.8)	8 (8.2)^a^
Secondary endpoints (MACE)	8 (7.5)	3 (3.1)

a P<0.05 for comparison between lipo-PGE_1_ and control. Data are presented as n (%). POM, perioperative mortality; MALE, major adverse limb events; MACE, major adverse cardiovascular events.

**Table III:** Cox proportional hazard regression model analysis for the primary study endpoint (combined POM and MALE).

Variable	Effect	HR	95% CI	P-value
Treatment group	Control vs. lipo-PGE_1_	2.15	1.01–4.57	<0.05
Class of ischemia	≥IIb vs. ≤IIa	3.61	1.37–9.51	<0.01
Surgical method	Bypass vs. TE	3.33	1.33–8.31	0.01

POM, perioperative mortality; MALE, major adverse limb events; HR, hazard ratio; CI, confidence interval; lipo-PGE_1_, liposomal prostaglandin E_1_; TE, thromboembolectomy.
